# Between‐study differences in grip strength: a comparison of Norwegian and Russian adults aged 40–69 years

**DOI:** 10.1002/jcsm.12816

**Published:** 2021-10-03

**Authors:** Rachel Cooper, Vladimir M. Shkolnikov, Alexander V. Kudryavtsev, Sofia Malyutina, Andrew Ryabikov, Laila Arnesdatter Hopstock, Jonas Johansson, Sarah Cook, David A. Leon, Bjørn Heine Strand

**Affiliations:** ^1^ Department of Sport and Exercise Sciences, Musculoskeletal Science and Sports Medicine Research Centre Manchester Metropolitan University Manchester UK; ^2^ International Laboratory for Population and Health National Research University Higher School of Economics Moscow Russia; ^3^ Laboratory of Demographic Data Max Planck Institute for Demographic Research Rostock Germany; ^4^ Northern State Medical University Arkhangelsk Russian Federation; ^5^ Department of Community Medicine UiT The Arctic University of Norway Tromsø Norway; ^6^ Research Institute of Internal and Preventive Medicine, Branch of Institute of Cytology and Genetics Siberian Branch of Russian Academy of Sciences Novosibirsk Russia; ^7^ Novosibirsk State Medical University Novosibirsk Russia; ^8^ Faculty of Epidemiology and Population Health London School of Hygiene & Tropical Medicine London UK; ^9^ National Heart and Lung Institute Imperial College London London UK; ^10^ Norwegian Institute of Public Health Oslo Norway; ^11^ Norwegian National Advisory Unit on Ageing and Health Vestfold Hospital Trust Tønsberg Norway; ^12^ Department of Geriatric Medicine Oslo University Hospital Oslo Norway

**Keywords:** Grip strength, Sarcopenia, Between‐country differences, Lifestyle, Body size

## Abstract

**Background:**

Identifying individuals with low grip strength is an initial step in many operational definitions of sarcopenia. As evidence indicates that contemporaneous Russian populations may have lower mean levels of grip strength than other populations in northern Europe, we aimed to: compare grip strength in Russian and Norwegian populations by age and sex; investigate whether height, body mass index, education, smoking status, alcohol use and health status explain observed differences and; examine implications for case‐finding low muscle strength.

**Methods:**

We used harmonized cross‐sectional data on grip strength and covariates for participants aged 40–69 years from the Russian Know Your Heart study (KYH) (*n* = 3833) and the seventh survey of the Norwegian Tromsø Study (*n* = 5598). Maximum grip strength (kg) was assessed using the same protocol and device in both studies. Grip strength by age, sex and study was modelled using linear regression and between‐study differences were predicted from these models. Sex‐specific age‐standardized differences in grip strength and in prevalence of low muscle strength were estimated using the European population standard of 2013.

**Results:**

Normal ranges of maximum grip strength in both studies combined were 33.8 to 67.0 kg in men and 18.7 to 40.1 kg in women. Mean grip strength was higher among Tromsø than KYH study participants and this difference did not vary markedly by age or sex. Adjustment for covariates, most notably height, attenuated between‐study differences but these differences were still evident at younger ages. For example, estimated between‐study differences in mean grip strength in fully adjusted models were 2.2 kg [95% confidence interval (CI) 1.4, 3.1] at 40 years and 1.0 kg (95% CI 0.5, 1.5) at 65 years in men (age × study interaction *P* = 0.09) and 1.1 kg (95% CI 0.4, 1.9) at age 40 years and −0.2 kg (95% CI −0.7, 0.3) at 65 years in women (age × study interaction *P* < 0.01).

**Conclusions:**

We found between‐study differences in mean grip strength that are likely to translate into greater future risk of sarcopenia and poorer prospects of healthy ageing for Russian than Norwegian study participants. For example, the average Russian participant had a similar level of grip strength to a Norwegian participant 7 years older. Our findings suggest these differences may have their origins in childhood highlighting the need to consider interventions in early life to prevent sarcopenia.

## Introduction

Age‐related declines in muscle function and mass pose a major threat to healthy ageing and the maintenance of independence in later life. When levels of muscle function and mass fall below clinical thresholds this is referred to as sarcopenia. The assignment of an ICD‐10 code^1^ to this ‘progressive and generalized skeletal muscle disorder’,^2^ which has major personal and societal impacts,^3^, ^4^ reflects growing recognition of its clinical importance.

Challenges remain in achieving the aim of a universally agreed, unified operational definition of sarcopenia.^5^ In 2018, the European Working Group on Sarcopenia in Older People (EWGSOP2), whose work has gained considerable attention, published an updated consensus on the definition and diagnosis of sarcopenia.^6^ According to EWGSOP2's recommendations, one of the initial steps in case‐finding sarcopenia is to identify individuals with low muscle strength. Where low muscle strength is identified, sarcopenia is considered probable, and in clinical practice, this is considered sufficient to trigger assessment of causes and initiate intervention. It is also only if low muscle strength is found that assessments of muscle quantity or quality are recommended. As the EWGSOP2 recommend applying universal cut‐points to grip strength to identify low muscle strength, any between‐country differences in grip strength would therefore be expected to impact on the prevalence of sarcopenia detected. Understanding the scale of between‐country differences in grip strength by age and sex and investigating the factors that may explain these differences is therefore important.

In a systematic review that synthesized published normative grip strength data from seven different UN regions, marked variations in grip strength across the life course were documented.^7^ These meta‐analyses showed that grip strength was generally higher in high‐income than in low‐income and middle‐income countries. Similar observations were made in the PURE study that assessed grip strength in adults aged 35 to 70 years residing in 21 countries in different world regions. In this study, the highest mean grip strength values were recorded in Europe and North America and the lowest in South and South East Asia and Africa.^8^ Differences in grip strength between countries in different world regions^7^, ^8^, ^9^, ^10^ are to be expected given major differences in lifetime exposures and body size and composition. However, it is noteworthy that a number of studies have also reported variation in grip strength between different populations within Europe.^11^, ^12^, ^13^, ^14^, ^15^ In one of these studies, participants of similar ages from Moscow, England, and Denmark were compared. The Russian participants were weaker than English and Danish participants at all ages up to 80 years. Despite these differences, associations of grip strength with mortality rate ratios were similar in all three settings.^14^ The explanation of differences in grip strength between contemporaneous Russian and other northern European populations, which may in part be the result of the very different mortality and health profiles and past unfavourable health experiences of Russians compared with most other Europeans,^16^, ^17^ requires further investigation. This is especially as the Russian sample analysed was from Moscow, which has a higher average level of educational attainment and income than Russians residing in other regions of the country.^14^

Using harmonized data from the Russian Know Your Heart (KYH) study and the seventh survey of the Norwegian Tromsø Study (Tromsø 7), we aimed to: compare grip strength by age and sex in Russian and Norwegian populations aged 40–69 years; investigate specific factors that may explain observed differences and; examine the implications of any observed differences in mean levels of grip strength for the case‐finding of low muscle strength.

## Methods

### Study populations

The KYH study is a cross‐sectional survey of men and women residing in two Russian cities; Novosibirsk (the third largest city in Russia with a population of ~1.5 million) and Arkhangelsk (with a population of ~350 000).^18^ The study population comprises men and women aged 35–69 years recruited from the general population using random sampling of addresses from regional health insurance databases. Participants were assessed during fieldwork conducted between November 2015 and January 2018. KYH included two study components; a baseline interview conducted at the study participant's home, and a health check at a primary care clinic, which included grip strength testing. After exclusion of invalid addresses and those addresses where no residents were of the anticipated age and sex, the percentage of participants invited who attended the health check was 66% in Arkhangelsk and 34% in Novosibirsk.^18^ A total of 5089 men and women participated in the baseline interview of whom 4504 attended the health check. Of these attendees, there were 3939 participants aged 40–69 years, and 3833 with valid grip strength measurements (106 did not participate in grip strength testing due to health problems or other reasons).

The Tromsø Study is a longitudinal study of men and women residing in the municipality of Tromsø, Norway (with a population of ~75 000). In the seventh survey (Tromsø 7), conducted between March 2015 and October 2016,^19^, ^20^ all Tromsø residents aged 40 years and older were invited to participate. In the first phase of Tromsø 7, a total of 21,083 (65% response) men and women participated in an assessment including questionnaires, interviews, biological sampling, and clinical examination of whom 17 646 were aged 40–69 years. A random sample of participants (*n* = 6,608 aged 40–69 years) were then invited to participate in a second phase of additional clinical assessments including grip strength. Of those participants aged 40–69 years included in the second phase, 5598 had valid grip strength measurements (seven did not participate in grip strength testing due to health problems or other reasons).

The KYH study received ethical approval from the ethics committees of the London School of Hygiene & Tropical Medicine (approval number 8808), Novosibirsk State Medical University (approval number 75), the Institute of Preventative Medicine, Novosibirsk, and the Northern State Medical University, Arkhangelsk (approval number 01/01‐15). Tromsø 7 was approved by the Regional Committee of Medical and Health Research Ethics (REC North approval number 2014/940) and the Norwegian Data Protection Authority. All participants in the KYH and Tromsø 7 studies provided written informed consent.

### Assessment of grip strength

Key aspects of the KYH study protocol were designed to be directly comparable with Tromsø 7.^18^ In both studies, grip strength was assessed by trained health professionals using Jamar+ Digital Dynamometers following the Southampton protocol.^21^ This involved participants being asked to sit in a chair holding the dynamometer while resting their arm on the chair's armrest with their elbow bent at a 90° angle and their hand positioned thumbs up. The same hand setting on the dynamometer was used for all participants. Participants received verbal encouragement from the tester to squeeze as hard as they could during each test. Three measurements were taken for each hand, alternating between right and left hands, and the maximum value achieved from all six measurements was used in analyses.

EWGSOP2 cut‐points were applied (grip strength: men <27 kg and women <16 kg) to identify those with low muscle strength.^6^ These cut‐points are based on a T score of −2.5 when using normative data from 12 British studies. As it has been suggested that a cut‐point of −2.5 may be too conservative, we also examined a cut‐point of −2.0 (men <32 kg and women <19 kg).^22^

### Covariates

In order to investigate potential explanations of any between‐study differences in grip strength observed, we selected a priori to study the following covariates: height; body mass index (BMI); education; smoking; alcohol use and; health status. These covariates had all been ascertained in comparable ways in the two studies. Height (m) and weight (kg) were measured, and BMI was calculated (kg/m^2^). Education was self‐reported and grouped into three categories: incomplete secondary, secondary, higher. Smoking status was categorized as never, ex or current smoker. Alcohol consumption was assessed using the Alcohol Use Disorders Identification Test (AUDIT), a 10‐item questionnaire with sum scores ranging from 0 to 40, where higher values indicate more harmful drinking.^23^ Three dichotomous health status variables were derived to indicate the presence or absence of self‐reported: (i) arthritis or osteoarthritis, (ii) myocardial infarction/heart attack or stroke, and (iii) diabetes.

### Statistical analyses

Estimation of grip strength [with 95% confidence intervals (CI)], and between‐study differences in grip strength were modelled using linear regression taking account of age and sex. To allow for variation in between‐study differences in grip strength by sex and age, the interaction terms sex by age by study, sex by age, sex by study, and study by age were included and from these models mean grip strengths by age, sex and study were predicted. We also reran models in men and women separately, which included age, study, and age by study interaction terms. In addition, for comparative purposes, mean grip strength was age‐standardized, using the direct method, in 5 year age bands (40–44, 45–49, …, 65–69) using the European population standard of 2013.^24^ These analyses included all 9431 participants (3833 in KYH and 5598 in Tromsø 7) with valid grip strength measures.

To investigate factors that may explain observed between‐study differences in grip strength, each covariate was added to the main linear regression model (i.e. a model including age, sex, and study and their interactions) by itself in turn before models were run in which height, then BMI, then all other covariates (i.e. education, smoking, alcohol use, and health status) were included. These models were all run on the sample with complete data on covariates [*n* = 8965 (3812 in KYH and 5153 in Tromsø 7)]—data were missing on BMI (*n* = 10 KYH, *n* = 14 Tromsø 7); height (*n* = 2 KYH, *n* = 12 Tromsø 7); alcohol use (*n* = 10 KYH, *n* = 377 Tromsø 7); smoking (*n* = 1 KYH, *n* = 38 Tromsø 7); education (*n* = 0 KYH, *n* = 60 Tromsø 7).

### Analyses of low grip strength

Sex‐specific age‐standardized prevalence of low muscle strength was estimated using the European population standard of 2013 for the full age range (40–69 years) as well as for the age range 60–69 years. Between‐study differences in age‐standardized prevalence of low muscle strength in men and women were estimated and tested in linear regression using the survey prefix (svy) command in Stata.

### Sensitivity analyses

As a 1 kg difference in grip strength represents a smaller proportion of a standard deviation in men than women, the main analyses were repeated using sex‐standardized grip strength values. These values were calculated as (*x*‐mean_*i*_)/SD_*j*_ where *x* is the observed measure of grip strength and mean*j* and SD*j* are the sample mean and SD of grip strength for sex *j*. To test the impact of restricting adjusted analyses to the sample with complete data on covariates, we reran basic models on a larger sample (*n* = 9407) who had only height and BMI data missing.

## Results

Characteristics of the two study populations are shown in *Table* 1. Tromsø 7 participants were on average older and taller and more likely to report higher levels of education and lower prevalence of specified health conditions than KYH participants. Mean BMI in the men from the two studies were similar, but differences were observed among women (28.9 kg/m^2^ in KYH vs. 26.7 kg/m^2^ in Tromsø 7) due to greater mean weight and also shorter mean height of women in KYH than Tromsø 7. Current smoking was more prevalent among KYH than Tromsø 7 participants and this difference was much more pronounced among men. Alcohol use scores were similar among men in the two studies. Although alcohol use scores in women from both studies were lower than those among men, women from Tromsø 7 had higher scores than women from KYH.

**Table 1:** Characteristics of the Know Your Heart (KYH) and Tromsø 7 study participants (*N* = 9431)^a^ included in the main analyses

	Descriptive statistics^b^			
	Men		Women	
	KYH	Tromsø 7	KYH	Tromsø 7
Age (years)	55.7 (8.3) 1622	58.6 (8.3) 2552	55.3 (8.4) 2211	58.3 (8.3) 3046
Height (cm)	174.9 (6.7) 1622	177.8 (6.7) 2548	161.1 (6.1) 2209	164.4 (6.3) 3038
Body mass index (kg/m^2^)	27.7 (4.8) 1619	27.9 (4.0) 2548	28.9 (6.2) 2204	26.7 (4.7) 3036
Education				
Incomplete secondary	141 (9)	581 (23)	124 (6)	713 (24)
Secondary	842 (52)	744 (29)	1209 (55)	842 (28)
Higher	639 (39)	1203 (48)	878 (40)	1455 (48)
Alcohol use score^c^	4 (2, 7) 1615	4 (3, 6) 2397	1 (1, 3) 2208	3 (2, 4) 2824
Smoking status				
Never	449 (28)	1001 (39)	1526 (69)	1129 (37)
Ex	588 (36)	1191 (47)	319 (14)	1453 (48)
Current	585 (36)	345 (14)	365 (17)	441 (15)
Myocardial infarction/Heart attack or stroke				
No	1431 (88)	2352 (92)	2065 (93)	2956 (97)
Yes	191 (12)	200 (8)	146 (7)	90 (3)
Arthritis or osteoarthritis				
No	1244 (77)	2183 (86)	1382 (63)	2197 (72)
Yes	378 (23)	369 (15)	829 (37)	849 (28)
Diabetes				
No	1497 (92)	2390 (94)	2000 (90)	2902 (95)
Yes	125 (8)	162 (6)	211 (10)	144 (5)

^a^ Maximum *N* = 9431. This includes all study participants aged 40–69 years with valid data on grip strength but Ns vary due to missing data on covariates.

^b^ Mean (SD) *N* for age, height and body mass index; median (q25, q75) *N* for alcohol use score; *N* (%) for all other variables.

^c^ Alcohol Use Disorders Identification Test (AUDIT) score with range 0–40, where higher values indicate more harmful drinking.

Normal ranges of maximum grip strength for both studies combined were 33.8 to 67.0 kg in men and 18.7 to 40.1 kg in women. These normal ranges varied by age group and study (supporting information *Table* S1).

**Figure 1:**
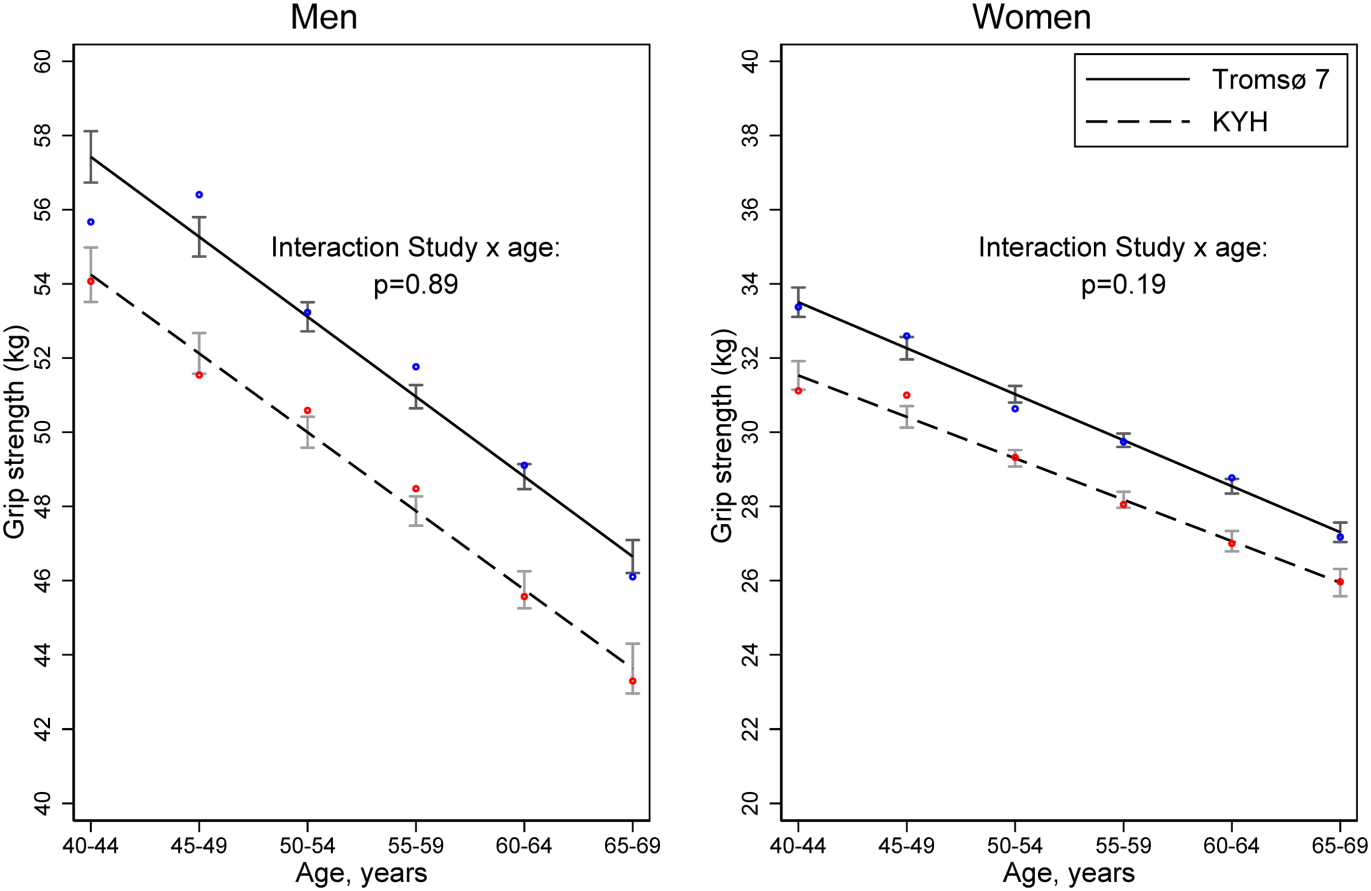
Mean grip strength (kg) (95% confidence intervals) by age and study in men and women. Estimated in sex‐specific linear regression models including age, study, and an age by study interaction (*N* = 9431). *Dots are unadjusted observed means (blue for Tromsø 7 and red for Know Your Heart Study)

In basic descriptive models, in all age groups and in both sexes, mean grip strength was higher among Tromsø 7 than KYH participants (refer to *Figure* 1 and *Table* S2). There was no evidence in these models that the differences in mean grip strength between studies varied by age in either sex (*P* values for age × study interactions in men = 0.89 and in women = 0.19) or that the study differences varied by sex (*P* value for study × sex × age interaction = 0.59).

**Figure 2:**
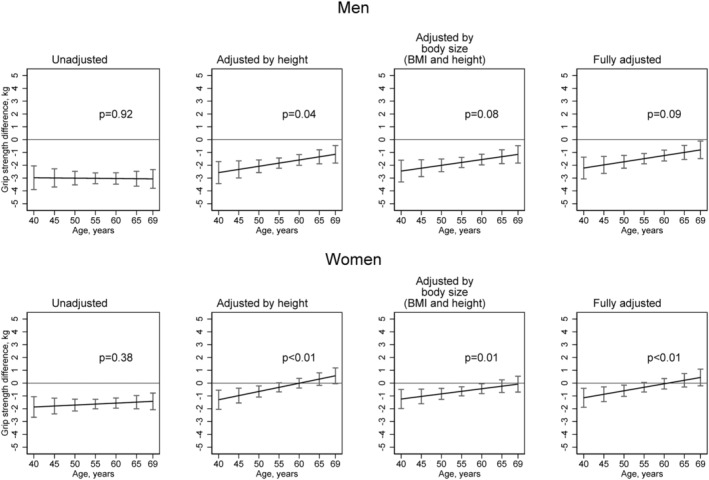
Absolute differences in mean grip strength (kg) (95% confidence intervals) between Know Your Heart and Tromsø 7 study participants by age and sex estimated in linear regression models with and without adjustments for covariates (Tromsø 7 is the reference line at 0) (*N* = 8965). Footnotes: In all models, age is modelled linearly including all its three‐ways and two‐ways interactions with study and sex. Fully adjusted model includes: eight, body mass index, education, smoking status, alcohol use and health status (indicated by presence or absence of self‐reported myocardial infarction/heart attack or stroke; arthritis or osteoarthritis; diabetes). *P* values presented are from tests of interaction between age and study.

Based on predicted estimates from a regression model including grip strength, age, sex, and study; and interaction terms between these three covariates, among men, mean grip strength was 3.0 kg (95% CI 2.1, 3.9) lower in KYH compared with Tromsø 7 participants at age 40 years, and 3.1 kg (95% CI 2.5, 3.6) lower at age 65 years (*Figure* 2). Adjustment for height attenuated between‐study differences, especially at older ages—in the model adjusted for height, estimated between study differences in mean grip strength were 2.6 kg (95% CI 1.7, 3.4) at 40 years and 1.3 kg (95% CI 0.8, 1.9) at 65 years. The addition of BMI had minimal impact, and in the fully adjusted model with inclusion of all covariates, differences in mean grip strength, though attenuated, were still observed at all ages—estimated between study differences in mean grip strength in this model were 2.2 kg (95% CI 1.4, 3.1) at 40 years and 1.0 kg (95% CI 0.5, 1.5) at 65 years (age × study interaction *P* = 0.09).

Among women, in the initial model, mean grip strength was 1.9 kg (95% CI 1.1, 2.7) lower in KYH compared with Tromsø 7 participants at age 40 years, and 1.5 kg (95% CI 1.0, 2.0) lower at age 65 years (*Figure* 2). Height substantially attenuated these between study differences, especially at older ages—in the model adjusted for height, estimated between study differences in mean grip strength were 1.3 kg (95% CI 0.5, 2.0) at 40 years and −0.3 kg (95% CI ‐0.8, 0.2) at 65 years. Thus, after adjustment for height, there was no evidence of a difference in mean grip strength between older KYH and Tromsø 7 women participants, whereas differences were still observed at younger ages. The addition of other covariates had only minimal impact and in the fully adjusted model estimated between study differences were 1.1 kg (95% CI 0.4, 1.9) at age 40 years and −0.2 kg (95% CI −0.7, 0.3) at 65 years (age × study interaction *P* < 0.01).

Although differences in mean grip strength between studies were observed across the full age‐range, there was limited evidence of differences in the age‐standardized prevalence of low muscle strength when the EWGSOP2 cut‐off points were applied to the full sample (*Table* 2). However, when estimated for older ages (i.e. 60–69 years) only, age‐standardized prevalence estimates of low muscle strength were higher in KYH compared with Tromsø 7 women (*Table* 2, *Figure* S1). Using a less conservative cut‐off point, the age‐standardized prevalence of low grip strength in men aged 40–69 years was 1.0% in Tromsø 7 and 1.1% in KYH (*P* = 0.76 for test of difference) and in women was 1.9% in Tromsø 7 and 2.8% in KYH (*P* = 0.03 for test of difference). These differences in prevalence were more pronounced when estimates were restricted to those aged 60–69 years [men: 1.8% in Tromsø 7 vs. 3.0% in KYH (*P* = 0.05 for test of difference) and women: 2.5% in Tromsø 7 vs. 6.0% in KYH (*P* < 0.001 for test of difference)].

**Table 2:** Prevalence (%) of low muscle strength by age, sex and study (using European Working Group on Sarcopenia in Older People (EWGSOP2) and alternative cut‐points^a^) (*N* = 9431)

Cut‐points	EWGSOP2 recommendation (<27 kg men, <16 kg women)				Less conservative alternative (<32 kg men, <19 kg women)			
	Men		Women		Men		Women	
Age (years)	KYH	Tromsø 7	KYH	Tromsø 7	KYH	Tromsø 7	KYH	Tromsø 7
40–44	0.0	0.0	0.0	0.7	0.5	0.0	1.5	0.7
45–49	0.0	0.0	0.0	0.3	0.4	0.0	0.3	0.9
50–54	0.0	0.8	0.8	1.2	0.7	1.6	2.7	2.7
55–59	0.7	0.0	0.0	0.7	1.1	0.3	3.8	2.1
60–64	0.6	0.8	1.2	0.4	2.7	1.5	5.6	1.3
65–69	1.9	0.5	2.5	0.7	3.3	2.1	6.4	3.7
Age‐standardized^b^ prevalence estimate for 40–69 years (95% CI)	0.4 (0.1, 0.6)	0.4 (0.2, 0.6)	0.5 (0.3, 0.7)	0.7 (0.4, 0.9)	1.1 (0.7, 1.6)	1.0 (0.7, 1.3)	2.8 (2.2, 3.3)	1.9 (1.5, 2.4)*
Age‐standardized^b^ prevalence estimate for 60–69 years (95% CI)	1.1 (0.5, 1.8)	0.7 (0.4, 1.0)	1.8 (1.1, 2.5)	0.5 (0.3, 0.8)*	3.0 (1.9, 4.0)	1.8 (1.3, 2.3)	6.0 (4.7, 7.3)	2.5 (1.9, 3.0)*

^a^ EWGSOP2^6^ recommended cut‐points for low muscle strength based on a *T* score of −2.5 when using normative grip strength data from 12 British studies^22^ the less conservative alternative applied is based on a *T* score of −2.0 using the same reference data.

^b^ Age‐standardized using the European population standard of 2013.

* Test of difference in age‐standardized prevalence estimate between Tromsø 7 and Know Your Heart study participants, *P* < 0.05. *P* values are calculated separately for men and women.

Findings were very similar when grip strength was sex‐standardized (*Figure* S2) with these results confirming that the scale of between‐study differences were similar in men and women and that the larger absolute between‐study differences in grip strength observed among men than women (refer to *Figures* 1 and 2) were attributable to the greater variance in grip strength among men. Findings were also very similar, and conclusions remained the same when analyses were rerun on a larger sample size (*Figure* S3).

## Discussion

Among a large contemporary sample of community‐dwelling men and women aged 40 to 69 years, we found clear and consistent evidence of weaker mean grip strength among Russian than Norwegian study participants. This translated into modest differences in the prevalence of low muscle strength in participants aged 60–69 years. Despite the presence of marked sex and age differences in grip strength in both studies as expected,^22^, ^25^ in basic models, there was no clear evidence that between‐study differences in grip strength varied by age or sex. Adjustment for covariates, specifically height, attenuated the observed between‐study differences at all ages and in both sexes but more so among older than younger participants and especially among women.

Our finding of weaker mean grip strength among Russian than Norwegian study participants is consistent with previous findings but provides a number of novel insights. In a comparison of the grip strength of men and women aged 55 to 89 years from three studies, participants from Moscow were found to be weaker on average than those from England and Denmark.^14^ The authors of this paper noted that differences in grip strength between Russian and other north European populations may be even larger than they had estimated because of the higher average levels of educational attainment and income among their Muscovite sample than the general Russian population. This is a potential limitation that we address by studying populations from two other regions of Russia selected to represent a range of different socio‐demographic levels.^18^ By including younger adults, our study is also able to provide other valuable new insights. Most importantly, it demonstrates that differences in grip strength between Russian and other north European populations are evident by age 40 years prior to the onset of declines in muscle function that are generally seen at older ages. This suggests a potentially important role for factors in earlier life that influence the development of peak muscle strength in explaining observed between‐study differences.

Consistent with the suggestion that early life factors are important is our finding that height, which has its origins in childhood,^26^ was the potential explanatory factor investigated that had the most marked impact on observed between‐study differences in grip strength. This is particularly notable given we also adjusted for markers of health status and health behaviours in adulthood. Because of the well‐documented adverse health profiles of Russian adults compared with other European populations,^16^, ^17^ we had expected that these factors, which precipitate age‐related declines in function, may be most important in explaining between‐study differences in grip strength.

That adjustment for height attenuated differences in grip strength to a greater extent among older than younger participants is explained by the smaller between‐study differences in mean height among younger than older participants. This is consistent with published data on secular trends in height showing that mean adult heights in Russia and Norway have converged over the course of the twentieth century.^27^ This is most likely because study participants from more recent born cohorts, especially those from Russia,^28^ have benefited more from improvements in early life conditions (including better nutrition and reduced risk of serious childhood infections) than those from older cohorts.

Our study has a number of key strengths that give us confidence that the between‐study differences in grip strength observed are real and very unlikely to be attributable to methodological artefacts. These include the use of the same model of dynamometer and identical grip strength measurement protocols. This is important given there are significant measurement differences in grip strength when different types of dynamometer and measurement protocols are employed.^29^, ^30^ In addition, we have been able to comprehensively investigate explanations of between‐study differences in grip strength due to the availability of data on a range of covariates assessed using comparable methods. However, we acknowledge that due to limitations in the variables available we may not have taken account of variation in all relevant aspects of health status. In addition, there are other factors we could not investigate that may explain differences in grip strength still observable in fully adjusted models at younger ages. Unfortunately, these other factors, including physical activity, fat, and lean mass and indicators of childhood development and circumstances, had not been assessed in one or both studies or had not been assessed comparably and so these warrant investigation in future studies.

Another strength of our study is the wide age range of participants. This allowed us to formally assess whether between‐study differences were observed from midlife and varied by age across 30 years of the adult life course. However, as these data are cross‐sectional, we cannot make inferences about rates of loss, and we recognize that observed differences in grip strength by age could be attributable to age, cohort, and/or period effects. As there was variation between studies in (i) levels of participation; (ii) the number of participants who had to be excluded as they were unable to complete the grip strength test for health reasons; (iii) the number of participants with missing data on covariates, we acknowledge that bias may have been introduced. However, KYH study participants were largely representative of the Russian urban population in terms of age and educational status when compared with the Russian 2010 census.^18^ As a greater number of KYH than Tromsø 7 participants were excluded from analyses as they could not complete the grip strength tests for health or other reasons (106 vs. 7) and these participants would be expected to have weaker strength,^31^ our estimates of between‐study differences are likely to be conservative. In addition, there was no evidence that exclusion of participants with missing data had a major impact as when we reran analyses on a less restricted sample, findings remained the same. Finally, we should note that the KYH dynamometers were sent to the UK for calibration post‐assessment at which point one of the four dynamometers used in Novosibirsk was found to be broken and systematically measuring 2.5 kg higher. We are unsure when the device was broken as it was not reported by any of the assessors and could have happened in transit back to the UK. We could find no evidence that any of the assessors recorded systematically higher grip strength measures. In addition, when we compared mean grip strength values in Tromsø 7 with KYH participants from Arkhangelsk only, our estimates of between‐study differences were very similar. We are therefore confident that this had no or very little impact on our findings and, as the error detected would have resulted in higher measurements among some Russian participants, if this had any impact, it would have led to more conservative estimates of between‐study differences.

The differences in mean grip strength observed in our analyses translated into modest between‐study differences in the prevalence of low muscle strength, as per EWGSOP2 and alternative criteria, at older ages only. That differences in the prevalence of low muscle strength were not detected at younger ages reflects the fact that before age 60, age‐related declines in grip strength would not yet have been expected to reach sufficient magnitude for many people to fall below proposed clinical thresholds. However, as the scale of between‐study differences in grip strength were very similar across all ages in basic models and these differences were not fully explained by adjustment for covariates in younger adults the implication of this is that Russian participants will be at greater risk of low muscle strength and hence sarcopenia in coming years as they continue to age and even if they experience similar rates of age‐related decline to Norwegian participants. To put the scale of the between‐study differences in perspective, we can use estimated differences in grip strength by age (which were 0.05 SD per year in men and 0.04 SD per year in women) and apply these to the overall age‐adjusted study differences in grip strength of 0.35 SD (95% CI 0.29, 0.41) in men and 0.30 SD (95% CI 0.25, 0.35) in women. When we do this, the between‐study differences in grip strength we observe correspond to an age difference of 7.0 years in men and 7.5 years in women.

In conclusion, we have shown that male and female study participants from Russia have consistently lower mean levels of grip strength than those from Norway between ages 40 and 69 years. As grip strength is commonly used to identify sarcopenia and has been proposed as a general biomarker of ageing,^32^, ^33^ these between‐study differences may translate into greater future risk of sarcopenia and poorer prospects of healthy ageing for Russian than Norwegian study participants. We also provide evidence to suggest that these differences may have their origins in early life supporting the need for life course approaches to the prevention of sarcopenia and promotion of healthy ageing. Strategies that promote muscle development in early life are likely to be an essential complement to those that minimize age‐related declines in later life.

## Conflicts of interest

None of the authors have any conflicts of interest to declare.

## Funding

S. M. and A. R. are supported by the Russian Academy of Science, State Target (АААА‐А17‐117112850280‐2). V. M. S. and D. A. L. were partly funded within the framework of the HSE University Basic Research Program.

## Ethical standards

The Know Your Heart study and Tromsø Study have both been approved by the appropriate ethics committee and have therefore been performed in accordance with the ethical standards laid down in the 1964 Declaration of Helsinki and its later amendments. All study participants gave their informed consent prior to their inclusion in the studies. Ethics committees in each location approved the use of these data for secondary analysis.

## Supporting information


**Table S1.** Normal ranges for maximum grip strength (kg) by age and sex for both studies combined and separately (*N* = 9,431).
**Table S2.** Mean grip strength (kg) by age and study in men and women estimated using linear regression models (N = 9,431).
**Figure S1.** Distribution of grip strength (kg) by age, sex and study. Two sets of cut‐points for low grip strength marked with dotted lines (those recommended by the European Working Group on Sarcopenia in Older People (EWGSOP2) (<27 kg for men, <16 kg for women) and less conservative values (<32 kg for men, <19 kg for women)).
**Figure S2.** Absolute differences in mean sex‐standardised grip strength (z‐scores) (95% confidence intervals) between Know Your Heart and Tromsø 7 study participants by age and sex estimated in linear regression models with and without adjustments for covariates (Tromsø 7 is the reference line at 0) (*N* = 8,965).
Note: Age is modelled linearly including all its 3‐and 2‐ways interactions with study and sex. Fully adjusted model includes: height, BMI, education, smoking status, alcohol use and health status (indicated by presence or absence of self‐reported myocardial infarction/heart attack or stroke; arthritis or osteoarthritis; diabetes). P‐values for interaction between age and study.
**Figure S3.** Absolute differences in mean grip strength (kg) (95% confidence intervals) between Know Your Heart and Tromsø 7 study participants by age and sex estimated in linear regression models with and without adjustment for height and BMI (Tromsø 7 is the reference line at 0).
**S**ensitivity analysis to compare unadjusted and height and BMI adjusted analyses run on main analytical sample with complete data on all covariates (*n* = 8,965) and a larger sample (*n* = 9,407) with complete data on height and BMI.
