# Supplementary material for: Between‐study differences in grip strength: a comparison of Norwegian and Russian adults aged 40–69 years
Source: J Cachexia Sarcopenia Muscle. 2021 Oct 3;12(6):2091–100. doi: 10.1002/jcsm.12816 (PMC8718040; doi:10.1002/jcsm.12816)
Supplement: Supplementary file 1 — Table S1. Normal ranges for maximum grip strength (kg) by age and sex for both studies combined and separately (N = 9,431). Table S2. Mean grip strength (kg) by age and study in men and women estimated using linear regression models (N = 9,431). Figure S1. Distribution of grip strength (kg) by age, sex and study. Two sets of cut‐points for low grip strength marked with dotted lines (those recommended by the European Working Group on Sarcopenia in Older People (EWGSOP2) (<27 kg for men, <16 kg for women) and less conservative values (<32 kg for men, <19 kg for women)). Figure S2. Absolute differences in mean sex‐standardised grip strength (z‐scores) (95% confidence intervals) between Know Your Heart and Tromsø 7 study participants by age and sex estimated in linear regression models with and without adjustments for covariates (Tromsø 7 is the reference line at 0) (N = 8,965). Note: Age is modelled linearly including all its 3‐and 2‐ways interactions with study and sex. Fully adjusted model includes: height, BMI, education, smoking status, alcohol use and health status (indicated by presence or absence of self‐reported myocardial infarction/heart attack or stroke; arthritis or osteoarthritis; diabetes). P‐values for interaction between age and study. Figure S3. Absolute differences in mean grip strength (kg) (95% confidence intervals) between Know Your Heart and Tromsø 7 study participants by age and sex estimated in linear regression models with and without adjustment for height and BMI (Tromsø 7 is the reference line at 0). Sensitivity analysis to compare unadjusted and height and BMI adjusted analyses run on main analytical sample with complete data on all covariates (n = 8,965) and a larger sample (n = 9,407) with complete data on height and BMI. [file JCSM-12-2091-s001.docx]

**Supporting Information for: Between-study differences in grip strength: a comparison of Norwegian and Russian adults aged 40-69 years**

**Supporting Information Table S1: Normal ranges for maximum grip strength (kg) by age and sex for both studies combined and separately (N=9,431)**

|  | **Both studies combined** | |  | **KYH** | |  | **Tromsø 7** | |
| --- | --- | --- | --- | --- | --- | --- | --- | --- |
| **Age (y)** | **2.5 centile** | **97.5 centile** |  | **2.5 centile** | **97.5 centile** |  | **2.5 centile** | **97.5 centile** |
| **Men** |  |  |  |  |  |  |  |  |
| 40-49 | 38.0 | 71.0 |  | 36.4 | 68.7 |  | 40.3 | 72.9 |
| 50-59 | 34.8 | 67.9 |  | 34.6 | 66.7 |  | 35.5 | 69.3 |
| 60-69 | 32.4 | 62.5 |  | 30.7 | 61.6 |  | 33.2 | 62.9 |
| Total (40-69) | 33.8 | 67.0 |  | 32.9 | 66.6 |  | 34.5 | 67.6 |
| **Women** |  |  |  |  |  |  |  |  |
| 40-49 | 21.4 | 43.4 |  | 21.0 | 42.6 |  | 22.8 | 44.4 |
| 50-59 | 18.6 | 40.1 |  | 17.8 | 39.2 |  | 19.2 | 40.6 |
| 60-69 | 17.7 | 37.1 |  | 16.5 | 36.2 |  | 18.9 | 37.3 |
| Total (40-69) | 18.7 | 40.1 |  | 17.7 | 40.1 |  | 19.5 | 40.2 |

KYH = Know Your Heart study

**Supporting Information Table S2: Mean grip strength (kg) by age and study in men and women estimated using linear regression models (N=9,431)**

|  | **Men** |  |  |  |  | **Women** | |  |  |
| --- | --- | --- | --- | --- | --- | --- | --- | --- | --- |
|  | **KYH** |  | **Tromsø 7** | |  | **KYH** |  | **Tromsø 7** | |
| **Age (y)** | **N** | **Mean (SD)** | **N** | **Mean (SD)** |  | **N** | **Mean (SD)** | **N** | **Mean (SD)** |
| 40-44 | 216 | 54.1 (7.8) | 231 | 55.7 (8.4) |  | 337 | 31.1 (5.6) | 281 | 33.4 (5.7) |
| 45-49 | 247 | 51.5 (8.1) | 263 | 56.4 (8.1) |  | 341 | 31.0 (5.3) | 316 | 32.6 (5.1) |
| 50-54 | 274 | 50.6 (8.4) | 251 | 53.2 (8.7) |  | 367 | 29.3 (5.2) | 330 | 30.6 (5.5) |
| 55-59 | 282 | 48.5 (8.0) | 319 | 51.8 (8.3) |  | 396 | 28.1 (5.0) | 429 | 29.7 (5.0) |
| 60-64 | 337 | 45.6 (7.7) | 743 | 49.1 (7.9) |  | 409 | 27.0 (5.1) | 835 | 28.8 (4.4) |
| 65-69 | 266 | 43.3 (7.1) | 745 | 46.1 (7.4) |  | 361 | 26.0 (5.1) | 855 | 27.2 (4.7) |
| Total | 1622 | 48.6 (8.6) | 2552 | 50.3 (8.7) |  | 2211 | 28.7 (5.5) | 3046 | 29.5 (5.3) |
|  | |  |  |  |  |  |  |  |  |
| Age-standardised mean grip strength (95% CI)^a^ | | 49.8 (49.5, 50.2) |  | 51.7 (51.4, 52.1) |  |  | 29.3 (29.1, 29.5) |  | 30.3 (30.1, 30.5) |

^a^ Age standardised using the European population standard of 2013
KYH = Know Your Heart study

**Supporting Information Figure S1: Distribution of grip strength (kg) by age, sex and study. Two sets of cut-points for low grip strength marked with dotted lines (those recommended by the European Working Group on Sarcopenia in Older People (EWGSOP2) (<27kg for men, <16kg for women) and less conservative values (<32kg for men, <19kg for women))**

**Supporting Information Figure S2: Absolute differences in mean sex-standardised grip strength (z-scores) (95% confidence intervals) between Know Your Heart and Tromsø 7 study participants by age and sex estimated in linear regression models with and without adjustments for covariates (Tromsø 7 is the reference line at 0) (N=8,965)**

Note: Age is modelled linearly including all its 3-and 2-ways interactions with study and sex. Fully adjusted model includes: height, BMI, education, smoking status, alcohol use and health status (indicated by presence or absence of self-reported myocardial infarction/heart attack or stroke; arthritis or osteoarthritis; diabetes). P-values for interaction between age and study.

**Supporting Information Figure S3: Absolute differences in mean grip strength (kg) (95% confidence intervals) between Know Your Heart and Tromsø 7 study participants by age and sex estimated in linear regression models with and without adjustment for height and BMI (Tromsø 7 is the reference line at 0).**

**S**ensitivity analysis to compare unadjusted and height and BMI adjusted analyses run on main analytical sample with complete data on all covariates (n=8,965) and a larger sample (n=9,407) with complete data on height and BMI
